# Incursion of Novel Highly Pathogenic Avian Influenza A(H5N8) Virus, the Netherlands, October 2020

**DOI:** 10.3201/eid2706.204464

**Published:** 2021-06

**Authors:** Nancy Beerens, Rene Heutink, Frank Harders, Marit Roose, Sylvia B.E. Pritz-Verschuren, Evelien A. Germeraad, Marc Engelsma

**Affiliations:** Wageningen Bioveterinary Research, Lelystad, the Netherlands

**Keywords:** incursion, influenza, influenza virus, viruses, highly pathogenic avian influenza A(H5N8) virus, HPAI, H5N8 subtype, genome sequence, evolution, swans, wild birds, the Netherlands

## Abstract

Highly pathogenic avian influenza A(H5N8) virus was detected in mute swans in the Netherlands during October 2020. The virus shares a common ancestor with clade 2.3.4.4b viruses detected in Egypt during 2018–2019 and has similar genetic composition. The virus is not directly related to H5N8 viruses from Europe detected in the first half of 2020.

Introduction of highly pathogenic avian influenza (HPAI) H5 clade 2.3.4.4 viruses in Europe caused substantial losses to the poultry industry during 2014–2020. Migratory waterfowl are implicated in the distribution of HPAI H5 viruses along flyways from breeding grounds in northern Russia to wintering sites in Europe (*1*–*3*). During 2016, clade 2.3.4.4b HPAI H5N8 viruses were introduced in Europe (*4*,*5*) and the Netherlands (*6*,*7*). More recent introductions of these viruses were detected in eastern Europe, Germany, and Bulgaria in the first half of 2020 (*8*,*9*).

On October 17, 2020, two mute swans (*Cygnus olor*) were found dead in the province of Utrecht, the Netherlands. The swans were diagnostically tested as part of the wild bird surveillance program for avian influenza virus. Swab samples from the trachea and cloaca were PCR-positive for avian influenza virus. The virus was subtyped as HPAI H5N8 and contained the hemagglutinin (HA) cleavage site sequence PLREKRRKR*GLF.

We performed full-genome sequencing as described (*6*) and classified the virus genetically as H5 clade 2.3.4.4b. We performed detailed phylogenetic analyses to study the origin of the novel H5N8 virus (A/mute_swan/Netherlands/20015931–001/2020, GISAID accession no. EPI591075; https://www.gisaid.org). For HA (Figure) and neuraminidase (NA) (Appendix 1 Figure 1), the closest genetic relative was isolated from a duck in Egypt during January 2019 (EPI399644; only HA/NA sequences are available). The virus also shares a common ancestor with other viruses detected in Egypt during 2018–2019 and with viruses detected in the Netherlands and Eurasia during 2016–2018. The HA and NA gene segments of the novel H5N8 virus do not cluster with the H5N8 viruses that caused widespread outbreaks in eastern Europe and Germany earlier in 2020 or with viruses detected in Bulgaria during 2020.

**Figure:**
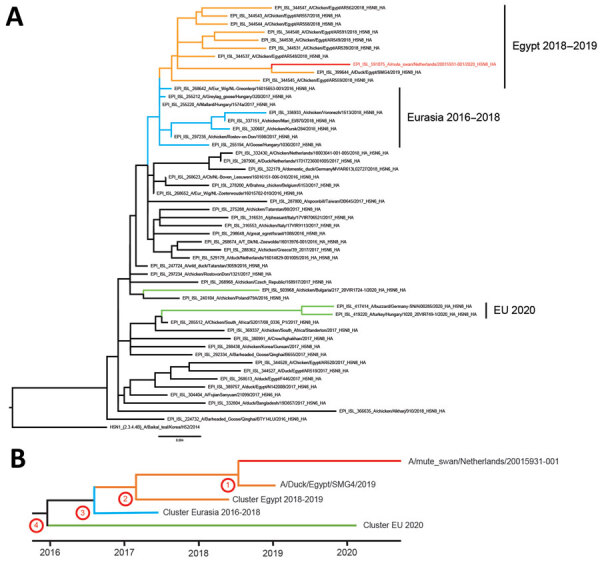
Phylogenetic analysis of the hemagglutinin (HA) segment of highly pathogenic avian influenza A(H5N8) virus, the Netherlands, October 2020. A) Optimal phylogenetic tree was generated by using the maximum-likelihood method (RAxML version 8.2.12; https://racm-ng.vital) with 100 bootstrap replicates and is shown and drawn to scale. GISAID (https://www.gisaid.org) accession numbers of the viruses are shown in the trees. Scale bar indicates nucleotide substitutions per site. B) Schematic representation of molecular dating of the HA gene segment. The Bayesian coalescent method was used to estimate the time to the most recent common ancestor of the novel H5N8 virus (numbers corresponding to nodes in the Table). Red branches indicate H5N8 virus isolated in the Netherlands in 2020;, green, H5N8 viruses isolated in eastern Europe, Germany, and Bulgaria in 2020; orange, viruses detected in Egypt during 2018–2019; and blue, viruses found in Eurasia during 2016–2018. EU, European Union.

For the other gene segments of the novel H5N8 virus, except for the matrix (M) protein segment, clustering was also observed with H5N8 viruses that circulated in Egypt during 2018–2019 and in Eurasia during 2016–2018 (Appendix 1 Figure 1). However, the M segment clusters with HPAI H5N8 viruses isolated in Asia and Egypt in 2016–2018 but also with the viruses found in eastern Europe and Germany during 2020, which suggests that reassortment with those viruses probably occurred for the M segment. No reassortments with low pathogenicity avian influenza viruses were observed for any of the segments. The genetic distance between the novel H5N8 virus and related viruses detected in Egypt and Eurasia appears relatively large, as demonstrated by the long branch lengths in phylogenetic trees (Appendix 1 Figure 1). This finding suggests long-term, undetected circulation of the virus or that intermediate virus sequences were not available in public databases.

We performed molecular dating by using BEAST (*10*) to estimate the time to the most recent common ancestor (Table; Appendix 1 Figure 2). For the H5 segment, a common ancestor of the novel H5N8 virus and the Egypt 2019 virus (accession no. EPI399644) was dated to July 2018 (node 1; Appendix 1 Figure 2) and with the cluster of viruses from Egypt to approximately March 2017 (node 2; Appendix 1 Figure 2). The common ancestor for the viruses from Eurasia detected during 2016–2018 was dated to August 2016 (node 3; Appendix 1 Figure 2) and with the viruses from eastern Europe and Germany detected in 2020 to approximately December 2015 (node 4; Appendix 1 Figure 2). Similar dating of ancestral viruses was observed for other gene segments, except for M (Appendix 1 Figure 2), for which the common ancestor for the viruses from eastern Europe and Germany detected during 2020 was dated to approximately May 2016 (node A; Appendix 1 Figure 2).

**Table:** Calculated tMRCA with 95% HPD and posterior value for highly pathogenic avian influenza A(H5N8) virus, the Netherlands, October 2020*

Segment	Node†	tMRCA		Height 95% HPD	Posterior value
		Year	Date		
PB2	1	ND	ND	ND	ND
	2	2016.67	Sep 2016	2016.43–2016.88	0.61
	3	2016.47	Jun 2016	2016.20–2016.68	0.97
	4	2012.70	Sep 2012	2010.50–2014.43	0.96
PB1	1	ND	ND	ND	ND
	2	2017.00	Jan 2017	2016.79–2017.14	0.95
	3	2016.56	Jul 2016	2016.35–2016.76	0.94
	4	2011.21	Mar 2011	2007.91–2013.81	1.00
PA	1	ND	ND	ND	ND
	2	2016.67	Sep 2016	2016.442016.88	0.01
	3	2016.48	Jun 2016	2016.30–2016.67	1.00
	4	2008.70	Sep 2008	2005.77–2011.20	1.00
HA	1	2018.58	Jul 2018	2018.15–2018.91	1.00
	2	2017.18	Mar 2017	2016.88–2017.44	1.00
	3	2016.62	Aug 2016	2016.46–2016.78	1.00
	4	2015.97	Dec 2015	2015.68–2016.23	0.97
NP	1	ND	ND	ND	ND
	2	2016.89	Nov 2016	2016.52–2017.13	0.87
	3	2016.43	Jun 2016	2016.08–2016.69	1.00
	4	2014.71	Sep 2014	2013.32–2015.77	0.95
NA	1	2018.42	Jun 2018	2017.87–2018.88	1.00
	2	2016.98	Dec 2016	2016.80–2017.12	0.99
	3	2016.71	Sep 2016	2016.51–2016.86	1.00
	4	2016.15	Feb 2016	2015.77–2016.40	1.00
M	A	2016.39	May 2016	2015.84–2016.63	0.19
NS	1	ND	ND	ND	ND
	2	2016.92	Dec 2016	2016.70–2017.03	0.01
	3	2016.48	Jun 2016	2016.00–2016.79	0.96
	4	2015.77	Oct 2015	2014.74–2016.40	1.00

*HA, hemagglutinin; HPD, highest posterior density interval; M, matrix protein; NA, neuraminidase; ND, not determined; NP, nucleoprotein; NS, nonstructural protein; PA, polymerase acidic, PB1, polymerase basic 1; PB2, polymerase basic 2; tMRCA, median time of the most recent common ancestor. †Nodes of the time-scaled phylogenetic tree.

Molecular dating analysis suggests that the ancestor of the novel H5N8 virus detected in the Netherlands during October 2020 has circulated in this genetic form since March 2017 and caused influenza outbreaks in Egypt during 2018–2019. The novel virus incursion is not related to viruses detected in eastern Europe, Germany, and Bulgaria earlier in 2020 but was probably associated with fall migration of wild birds to wintering sites in the Netherlands. Although no HPAI viruses or deaths were observed at wild bird breeding sites in northern Russia, HPAI H5N8 viruses were reported in southern Russia and northern Kazakhstan in September 2020. Some waterfowl species, such as Eurasian wigeon (*Anas penelope*), tufted duck (*Aythya fuligula*), and white-fronted goose (*Anser albifrons*), are known to migrate from these regions to the Netherlands (Dutch Centre For Field Ornithology, https://vogeltrekatlas.nl/soortzoek2.html).

The novel virus was first detected in 2 mute swans that do not migrate over long distances. However, a few days later, virus was also detected in a dead Eurasian wigeon, suggesting that this bird species might have been involved in the incursion of the virus into the Netherlands. Because sequences of the viruses detected in Russia and Kazakhstan are unknown, the relationship between these viruses and the virus detected in the Netherlands remains to be determined. During October, wild bird migration is ongoing, and millions of wild birds will reach their wintering sites in Europe in the coming months. This early detection of HPAI H5N8 virus in the Netherlands predicted a high risk for the poultry industry in Europe during the 2020–2021 winter season.

Appendix 1 Additional information on incursion of novel highly pathogenic avian influenza A(H5N8) virus, the Netherlands, October 2020.

Appendix 2 Authors and submitting laboratories who provided sequences from the GISAID EpiFlu Database used in study of incursion of novel highly pathogenic avian influenza A(H5N8) virus, the Netherlands, October 2020.
