# Supplementary material for: Incursion of Novel Highly Pathogenic Avian Influenza A(H5N8) Virus, the Netherlands, October 2020
Source: Emerg Infect Dis. 2021 Jun;27(6):1750–3. doi: 10.3201/eid2706.204464 (PMC8153856; doi:10.3201/eid2706.204464)
Supplement: Appendix 1 — Additional information on incursion of novel highly pathogenic avian influenza A(H5N8) virus, the Netherlands, October 2020. [file 20-4464-Techapp-s1.pdf]

Article DOI: https://doi.org/10.3201/eid2706.204464

Incursion of Novel Highly Pathogenic Avian Influenza A(H5N8) Virus, the Netherlands, October 2020

Appendix

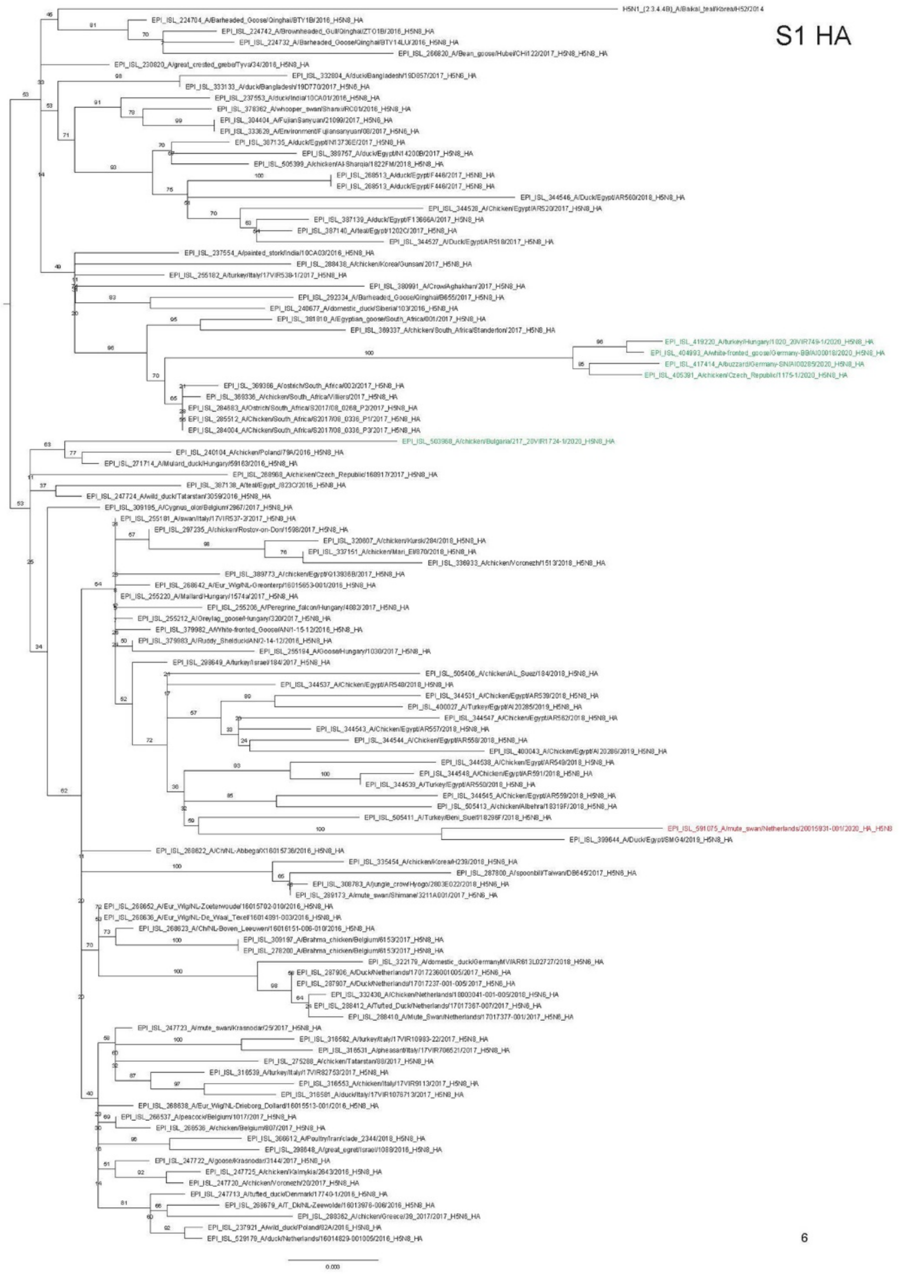

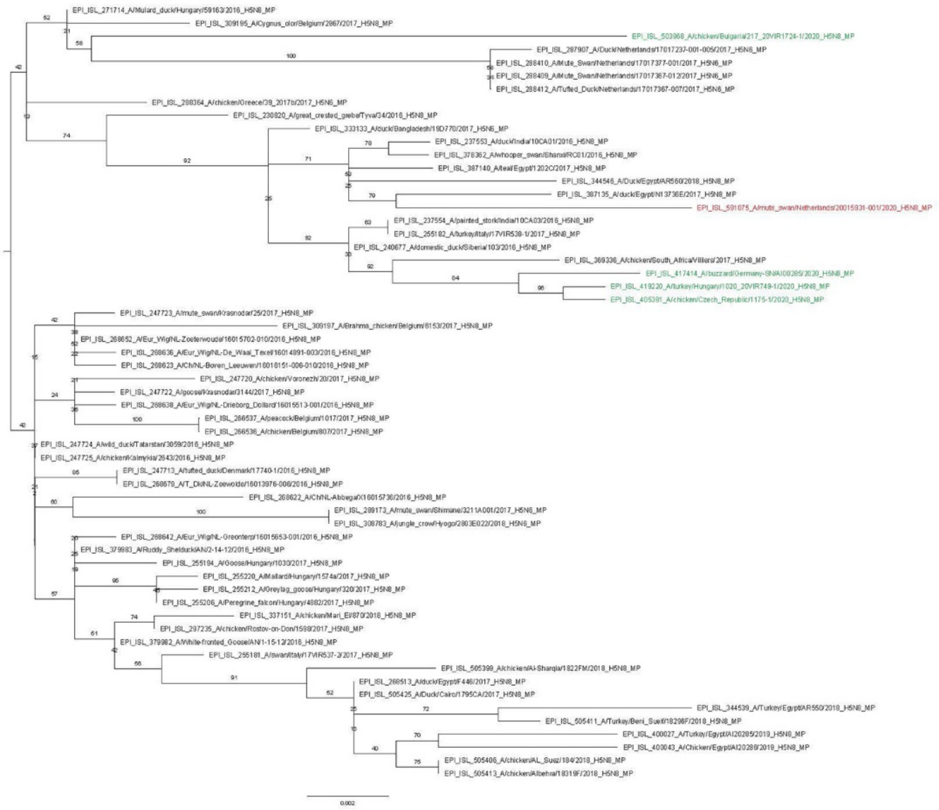

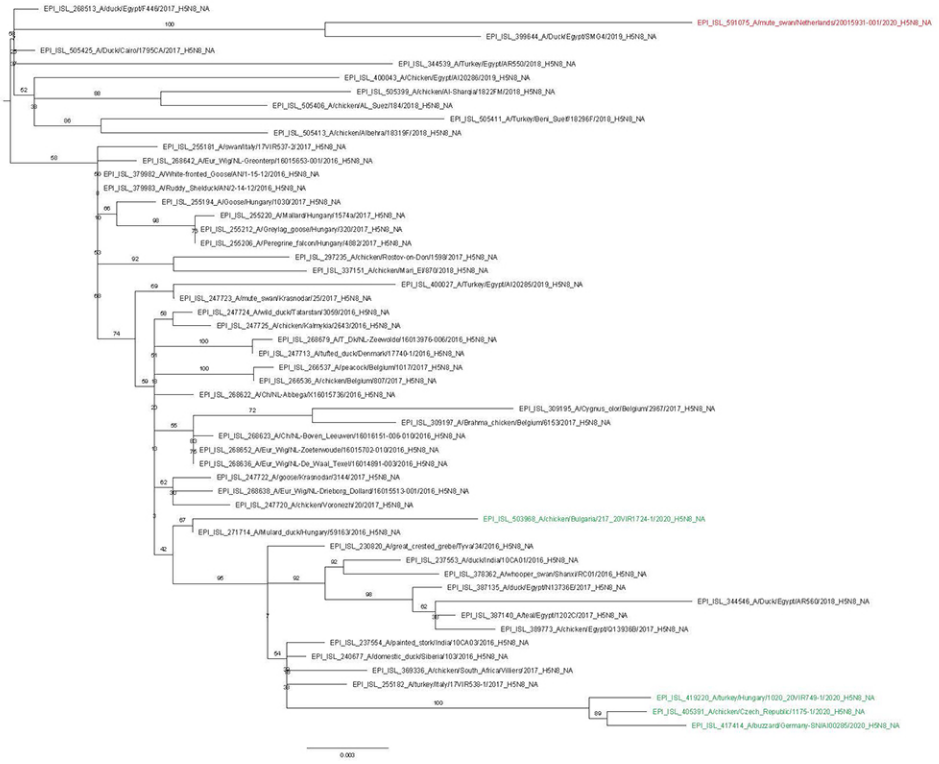

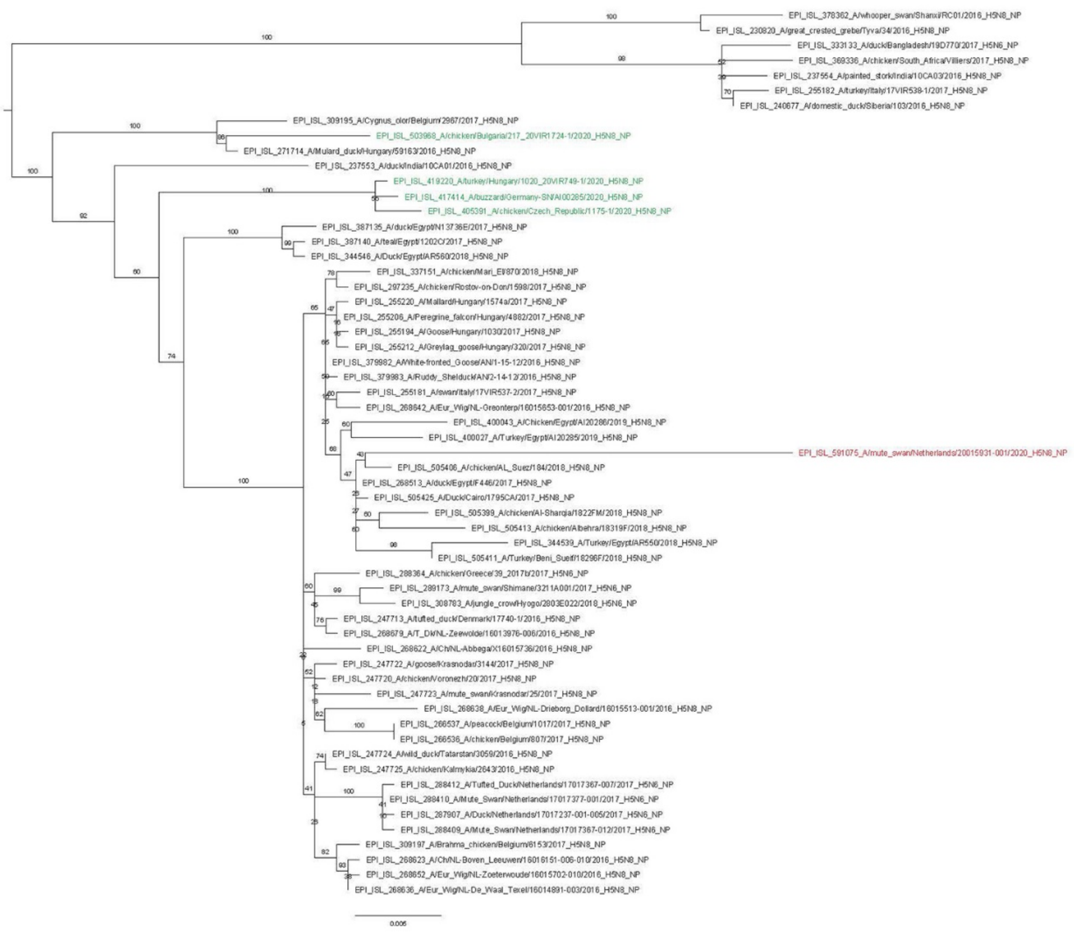

EPI_ISL_378362_A/whooper_swan/Shanxi/RC01/2016_H5N8_NP
EPI_ISL_230820_A/great_crested_grebe/Tyva/34/2016_H5N8_NP
EPI_ISL_333133_A/duck/Bangladesh/19D770/2017_H5N6_NP
EPI_ISL_369336_A/chicken/South_Africa/Villiers/2017_H5N8_NP
EPI_ISL_237554_A/painted_stork/India/10CA03/2016_H5N8_NP
EPI_ISL_255182_A/turkey/Italy/17VIR538-1/2017_H5N8_NP
EPI_ISL_240677_A/domestic_duck/Siberia/103/2016_H5N8_NP
EPI_ISL_309195_A/Cygnus_olor/Belgium/2967/2017_H5N8_NP
EPI_ISL_503968_A/chicken/Bulgaria/217_20VIR1724-1/2020_H5N8_NP
EPI_ISL_271714_A/Mulard_duck/Hungary/59163/2016_H5N8_NP
EPI_ISL_237553_A/duck/India/10CA01/2016_H5N8_NP
EPI_ISL_419220_A/turkey/Hungary/1020_20VIR749-1/2020_H5N8_NP
EPI_ISL_417414_A/buzzard/Germany-SN/AI00285/2020_H5N8_NP
EPI_ISL_405391_A/chicken/Czech_Republic/1175-1/2020_H5N8_NP
EPI_ISL_387135_A/duck/Egypt/N13736E/2017_H5N8_NP
EPI_ISL_387140_A/teal/Egypt/1202C/2017_H5N8_NP
EPI_ISL_344546_A/Duck/Egypt/AR560/2018_H5N8_NP
EPI_ISL_337151_A/chicken/Mari_El/870/2018_H5N8_NP
EPI_ISL_297235_A/chicken/Rostov-on-Don/1598/2017_H5N8_NP
EPI_ISL_255220_A/Mallard/Hungary/1574a/2017_H5N8_NP
EPI_ISL_255206_A/Peregrine_falcon/Hungary/4882/2017_H5N8_NP
EPI_ISL_255194_A/Goose/Hungary/1030/2017_H5N8_NP
EPI_ISL_255212_A/Greylag_goose/Hungary/320/2017_H5N8_NP
EPI_ISL_379982_A/White-fronted_Goose/AN/1-15-12/2016_H5N8_NP
EPI_ISL_379983_A/Ruddy_Shelduck/AN/2-14-12/2016_H5N8_NP
EPI_ISL_255181_A/swan/Italy/17VIR537-2/2017_H5N8_NP
EPI_ISL_268642_A/Eur_Wig/NL-Greonterp/16015653-001/2016_H5N8_NP
EPI_ISL_400043_A/Chicken/Egypt/AI20286/2019_H5N8_NP
EPI_ISL_400027_A/Turkey/Egypt/AI20285/2019_H5N8_NP
EPI_ISL_591075_A/mute_swan/Netherlands/20015931-001/2020_H5N8_NP
EPI_ISL_505406_A/chicken/AL_Suez/184/2018_H5N8_NP
EPI_ISL_268513_A/duck/Egypt/F446/2017_H5N8_NP
EPI_ISL_505425_A/Duck/Cairo/1795CA/2017_H5N8_NP
EPI_ISL_505399_A/chicken/Al-Sharqia/1822FM/2018_H5N8_NP
EPI_ISL_505413_A/chicken/Albehra/18319F/2018_H5N8_NP
EPI_ISL_344539_A/Turkey/Egypt/AR550/2018_H5N8_NP
EPI_ISL_505411_A/Turkey/Beni_Sueif/18296F/2018_H5N8_NP
EPI_ISL_288364_A/chicken/Greece/39_2017b/2017_H5N6_NP
EPI_ISL_289173_A/mute_swan/Shimane/3211A001/2017_H5N6_NP
EPI_ISL_308783_A/jungle_crow/Hyogo/2803E022/2018_H5N6_NP
EPI_ISL_247713_A/tufted_duck/Denmark/17740-1/2016_H5N8_NP
EPI_ISL_268679_A/T_Dk/NL-Zeewolde/16013976-006/2016_H5N8_NP
EPI_ISL_268622_A/Ch/NL-Abbega/X16015736/2016_H5N8_NP
EPI_ISL_247722_A/goose/Krasnodar/3144/2017_H5N8_NP
EPI_ISL_247720_A/chicken/Voronezh/20/2017_H5N8_NP
EPI_ISL_247723_A/mute_swan/Krasnodar/25/2017_H5N8_NP
EPI_ISL_268638_A/Eur_Wig/NL-Drieborg_Dollard/16015513-001/2016_H5N8_NP
EPI_ISL_266537_A/peacock/Belgium/1017/2017_H5N8_NP
EPI_ISL_266536_A/chicken/Belgium/807/2017_H5N8_NP
EPI_ISL_247724_A/wild_duck/Tatarstan/3059/2016_H5N8_NP
EPI_ISL_247725_A/chicken/Kalmykia/2643/2016_H5N8_NP
EPI_ISL_288412_A/Tufted_Duck/Netherlands/17017367-007/2017_H5N6_NP
EPI_ISL_288410_A/Mute_Swan/Netherlands/17017377-001/2017_H5N6_NP
EPI_ISL_287907_A/Duck/Netherlands/17017237-001-005/2017_H5N6_NP
EPI_ISL_288409_A/Mute_Swan/Netherlands/17017367-012/2017_H5N6_NP
EPI_ISL_309197_A/Brahma_chicken/Belgium/6153/2017_H5N8_NP
EPI_ISL_268623_A/Ch/NL-Boven_Leeuwen/16016151-006-010/2016_H5N8_NP
EPI_ISL_268652_A/Eur_Wig/NL-Zoeterwoude/16015702-010/2016_H5N8_NP
EPI_ISL_268636_A/Eur_Wig/NL-De_Waal_Texel/16014891-003/2016_H5N8_NP
0.005

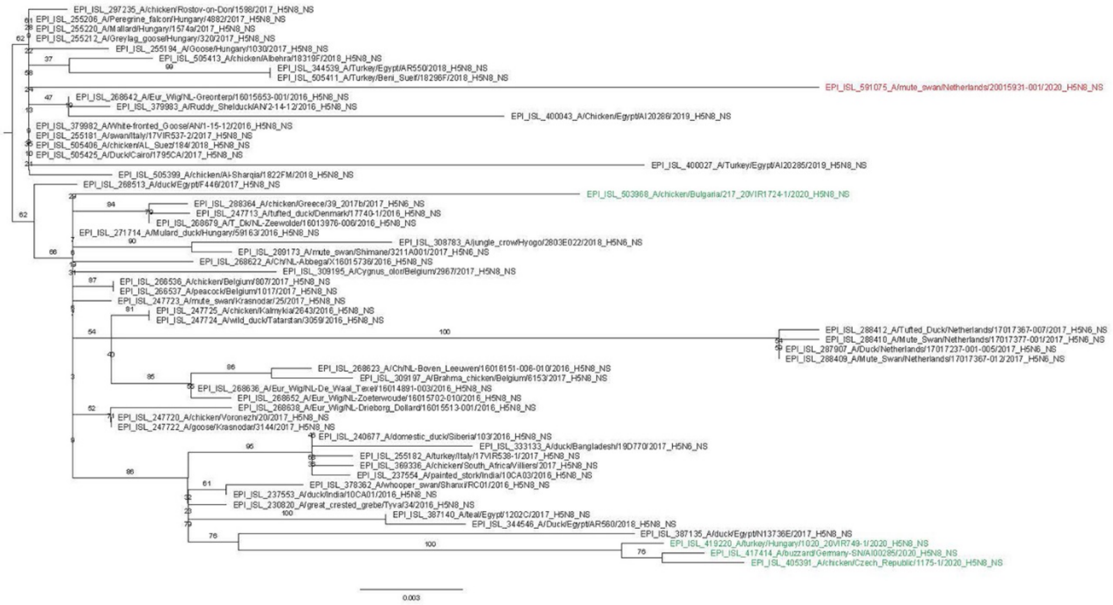

EPI_ISL_297235_A/chicken/Rostov-on-Don/1598/2017_H5N8_NS
EPI_ISL_255206_A/Peregrine_falcon/Hungary/4882/2017_H5N8_NS
EPI_ISL_255220_A/Mallard/Hungary/1574a/2017_H5N8_NS
EPI_ISL_255212_A/Greylag_goose/Hungary/320/2017_H5N8_NS
EPI_ISL_255194_A/Goose/Hungary/1030/2017_H5N8_NS
EPI_ISL_505413_A/chicken/Albehra/18319F/2018_H5N8_NS
EPI_ISL_344539_A/Turkey/Egypt/AR550/2018_H5N8_NS
EPI_ISL_505411_A/Turkey/Beni_Sueif/18296F/2018_H5N8_NS
EPI_ISL_591075_A/mute_swan/Netherlands/20015931-001/2020_H5N8_NS
EPI_ISL_268642_A/Eur_Wig/NL-Greonterp/16015653-001/2016_H5N8_NS
EPI_ISL_379983_A/Ruddy_Shelduck/AN/2-14-12/2016_H5N8_NS
EPI_ISL_400043_A/Chicken/Egypt/AI20286/2019_H5N8_NS
EPI_ISL_379982_A/White-fronted_Goose/AN/1-15-12/2016_H5N8_NS
EPI_ISL_255181_A/swan/Italy/17VIR537-2/2017_H5N8_NS
EPI_ISL_505406_A/chicken/AL_Suez/184/2018_H5N8_NS
EPI_ISL_505425_A/Duck/Cairo/1795CA/2017_H5N8_NS
EPI_ISL_400027_A/Turkey/Egypt/AI20285/2019_H5N8_NS
EPI_ISL_505399_A/chicken/Al-Sharqia/1822FM/2018_H5N8_NS
EPI_ISL_268513_A/duck/Egypt/F446/2017_H5N8_NS
EPI_ISL_503968_A/chicken/Bulgaria/217_20VIR1724-1/2020_H5N8_NS
EPI_ISL_288364_A/chicken/Greece/39_2017b/2017_H5N6_NS
EPI_ISL_247713_A/tufted_duck/Denmark/17740-1/2016_H5N8_NS
EPI_ISL_268679_A/T_Dk/NL-Zeewolde/16013976-006/2016_H5N8_NS
EPI_ISL_271714_A/Mulard_duck/Hungary/59163/2016_H5N8_NS
EPI_ISL_308783_A/jungle_crow/Hyogo/2803E022/2018_H5N6_NS
EPI_ISL_289173_A/mute_swan/Shimane/3211A001/2017_H5N6_NS
EPI_ISL_268622_A/Ch/NL-Abbega/X16015736/2016_H5N8_NS
EPI_ISL_309195_A/Cygnus_olor/Belgium/2967/2017_H5N8_NS
EPI_ISL_266536_A/chicken/Belgium/807/2017_H5N8_NS
EPI_ISL_266537_A/peacock/Belgium/1017/2017_H5N8_NS
EPI_ISL_247723_A/mute_swan/Krasnodar/25/2017_H5N8_NS
EPI_ISL_247725_A/chicken/Kalmykia/2643/2016_H5N8_NS
EPI_ISL_247724_A/wild_duck/Tatarstan/3059/2016_H5N8_NS
EPI_ISL_288412_A/Tufted_Duck/Netherlands/17017367-007/2017_H5N6_NS
EPI_ISL_288410_A/Mute_Swan/Netherlands/17017377-001/2017_H5N6_NS
EPI_ISL_287907_A/Duck/Netherlands/17017237-001-005/2017_H5N6_NS
EPI_ISL_288409_A/Mute_Swan/Netherlands/17017367-012/2017_H5N6_NS
EPI_ISL_268623_A/Ch/NL-Boven_Leeuwen/16016151-006-010/2016_H5N8_NS
EPI_ISL_309197_A/Brahma_chicken/Belgium/6153/2017_H5N8_NS
EPI_ISL_268636_A/Eur_Wig/NL-De_Waal_Texel/16014891-003/2016_H5N8_NS
EPI_ISL_268652_A/Eur_Wig/NL-Zoeterwoude/16015702-010/2016_H5N8_NS
EPI_ISL_268638_A/Eur_Wig/NL-Drieborg_Dollard/16015513-001/2016_H5N8_NS
EPI_ISL_247720_A/chicken/Voronezh/20/2017_H5N8_NS
EPI_ISL_247722_A/goose/Krasnodar/3144/2017_H5N8_NS
EPI_ISL_240677_A/domestic_duck/Siberia/103/2016_H5N8_NS
EPI_ISL_333133_A/duck/Bangladesh/19D770/2017_H5N6_NS
EPI_ISL_255182_A/turkey/Italy/17VIR538-1/2017_H5N8_NS
EPI_ISL_369336_A/chicken/South_Africa/Villiers/2017_H5N8_NS
EPI_ISL_237554_A/painted_stork/India/10CA03/2016_H5N8_NS
EPI_ISL_378362_A/whooper_swan/Shanxi/RC01/2016_H5N8_NS
EPI_ISL_237553_A/duck/India/10CA01/2016_H5N8_NS
EPI_ISL_230820_A/great_crested_grebe/Tyva/34/2016_H5N8_NS
EPI_ISL_387140_A/teal/Egypt/1202C/2017_H5N8_NS
EPI_ISL_344546_A/Duck/Egypt/AR560/2018_H5N8_NS
EPI_ISL_387135_A/duck/Egypt/N13736E/2017_H5N8_NS
EPI_ISL_419220_A/turkey/Hungary/1020_20VIR749-1/2020_H5N8_NS
EPI_ISL_417414_A/buzzard/Germany-SN/AI00285/2020_H5N8_NS
EPI_ISL_405391_A/chicken/Czech_Republic/1175-1/2020_H5N8_NS
0.003

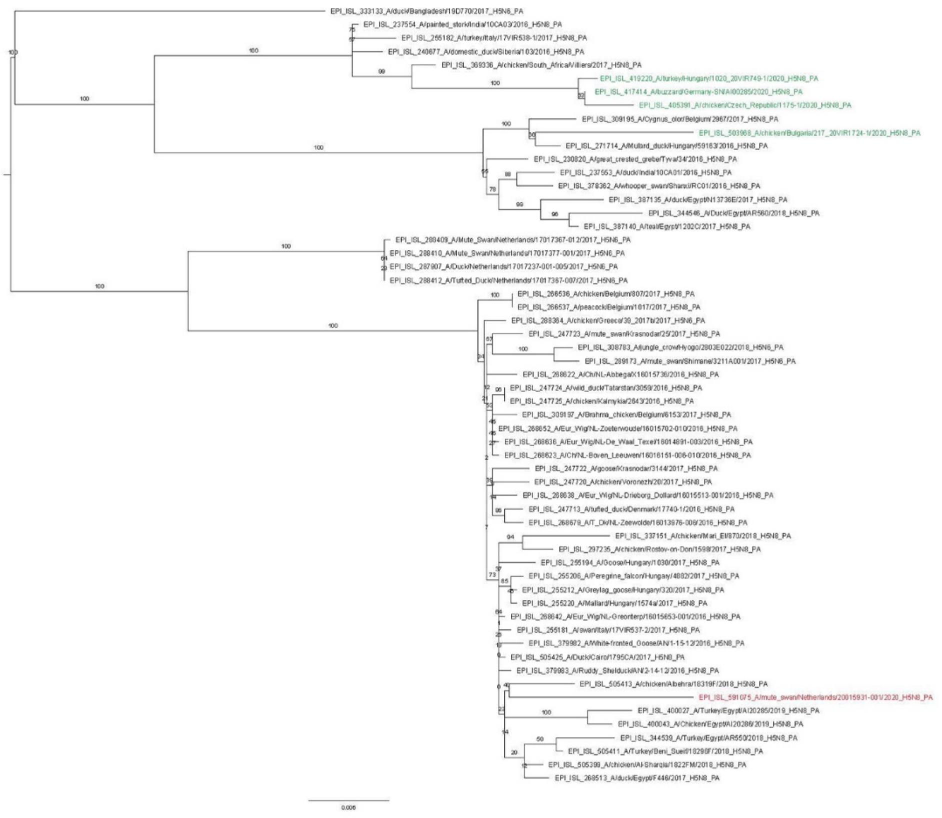

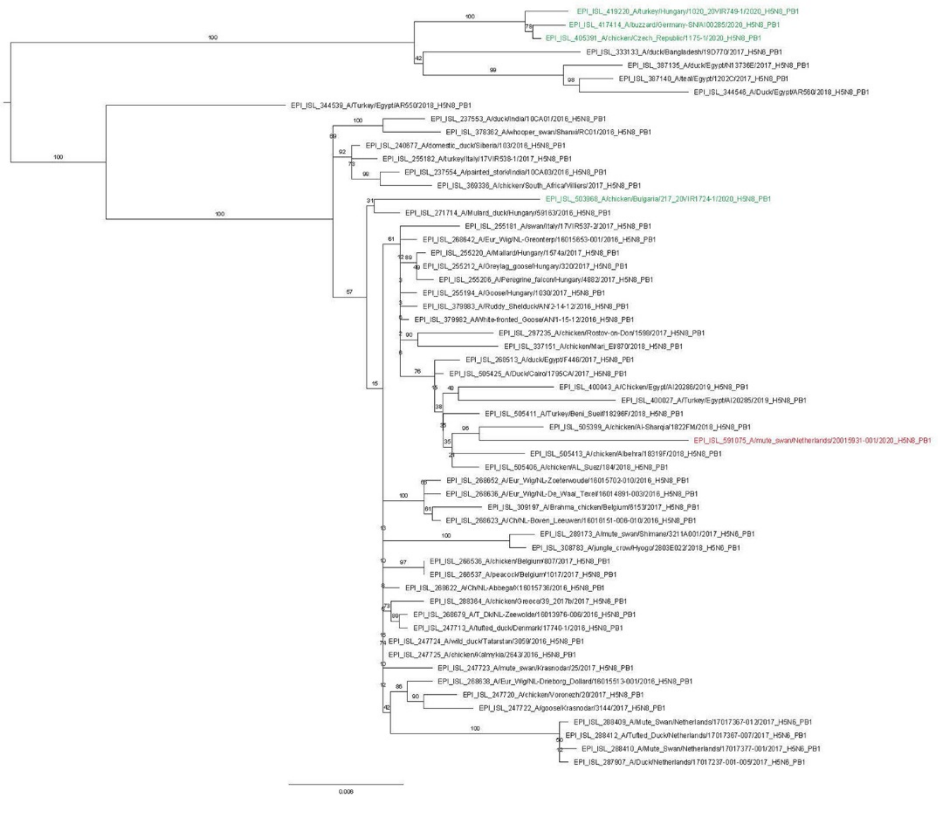

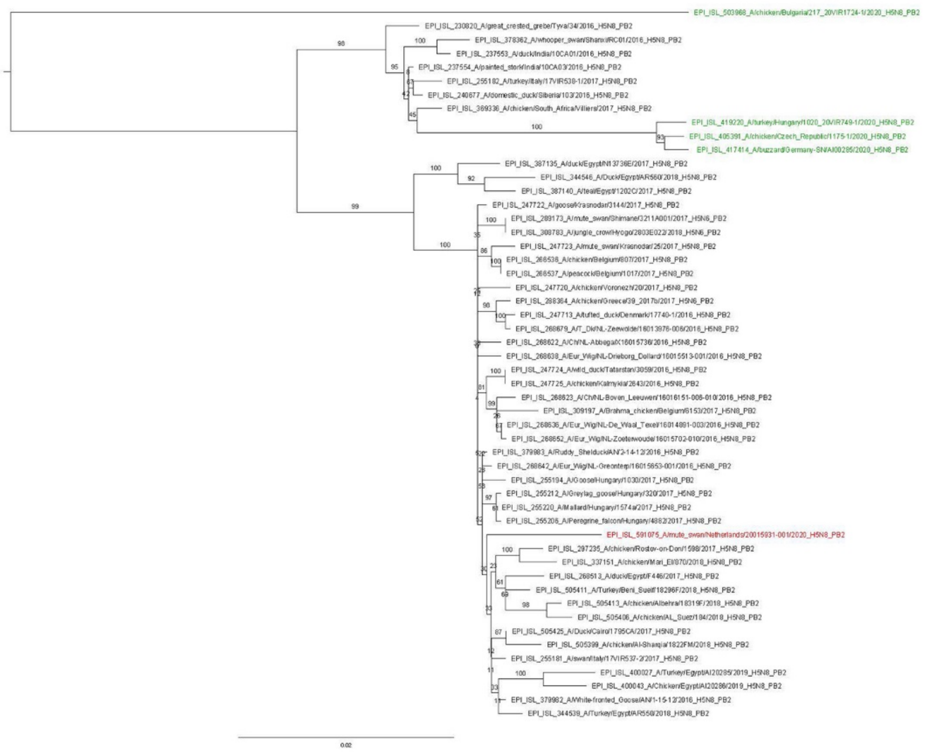

Appendix Figure 1. Related sequences were obtained from the GISAID EpiFlu database on October 21, 2020 (http://www.gisaid.org) (*1*) by using a BLAST search. For HA, additional sequences for H5 clade 2.3.4.4b were collected from the EpiFlu database and clustered by using the CD-HIT algorithm (*2*) and an identity setting of 0.985. Cluster representatives were used in the analysis of HA, in addition to the related sequences from the GISAID BLAST search. Sequences were aligned by using MAFFT v7.427 (*3*). Maximum-likelihood trees based on the general time-reversible model with a gamma-distributed variation of rates, and 100 bootstraps were generated by using RAxML v8.2.12 (*4*). The GISAID accession numbers of the viruses are shown in the trees (Appendix Table). The H5N8 virus isolated in the Netherlands in 2020 is marked in red, the H5N8 viruses isolated in Eastern-Europe, Germany and Bulgaria in 2020 are marked in green. Scale bars indicate nucleotide substitutions per site. HA, hemagglutinin; MP, matrix protein; NA, neuraminidase; NP, nucleoprotein; NS, nonstructural protein; PA, polymerase acidic, PB1, polymerase basic 1; PB2, polymerase basic 2.

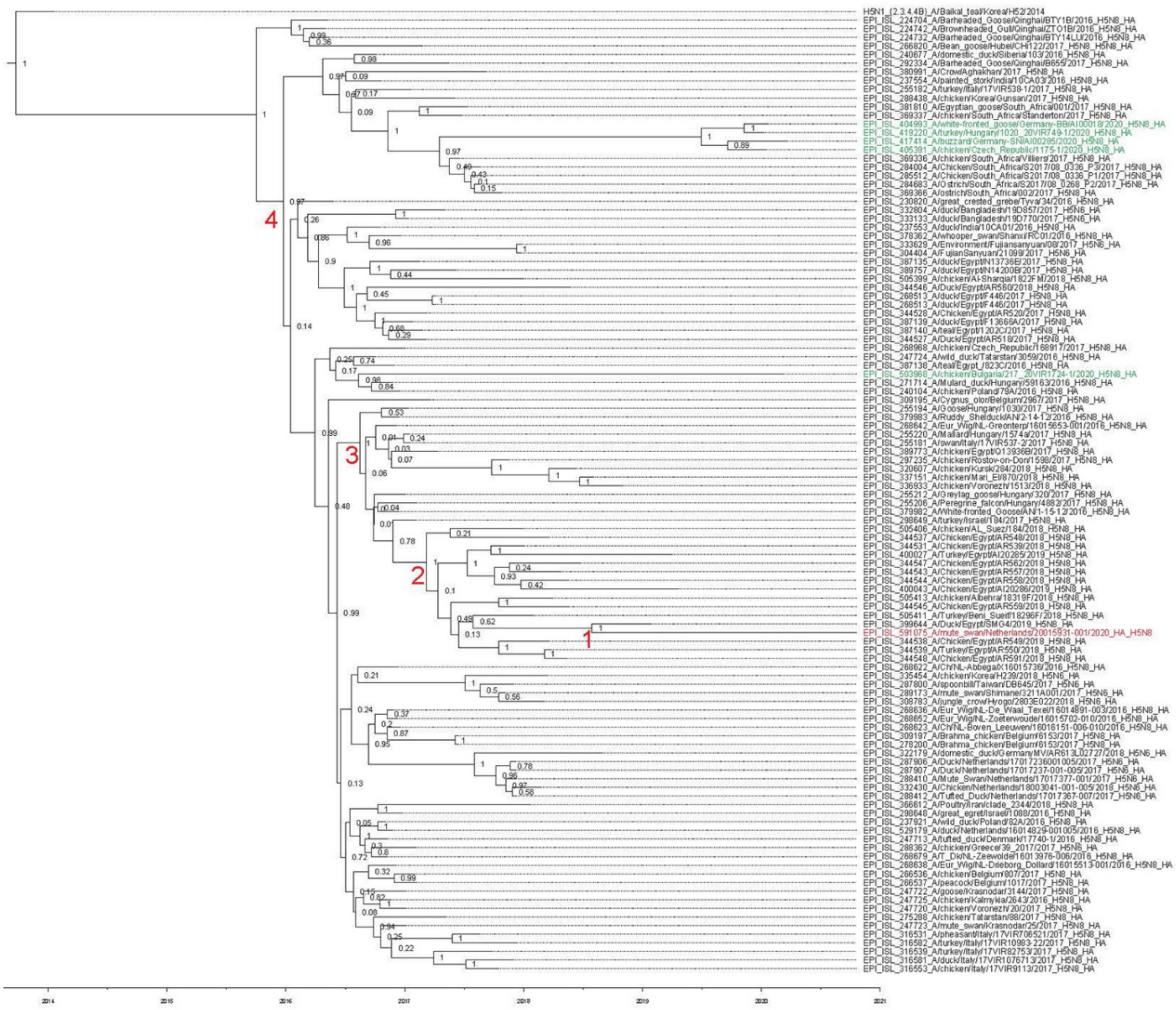
4
3
2
1
2014
2015
2016
2017
2018
2019
2020
2021

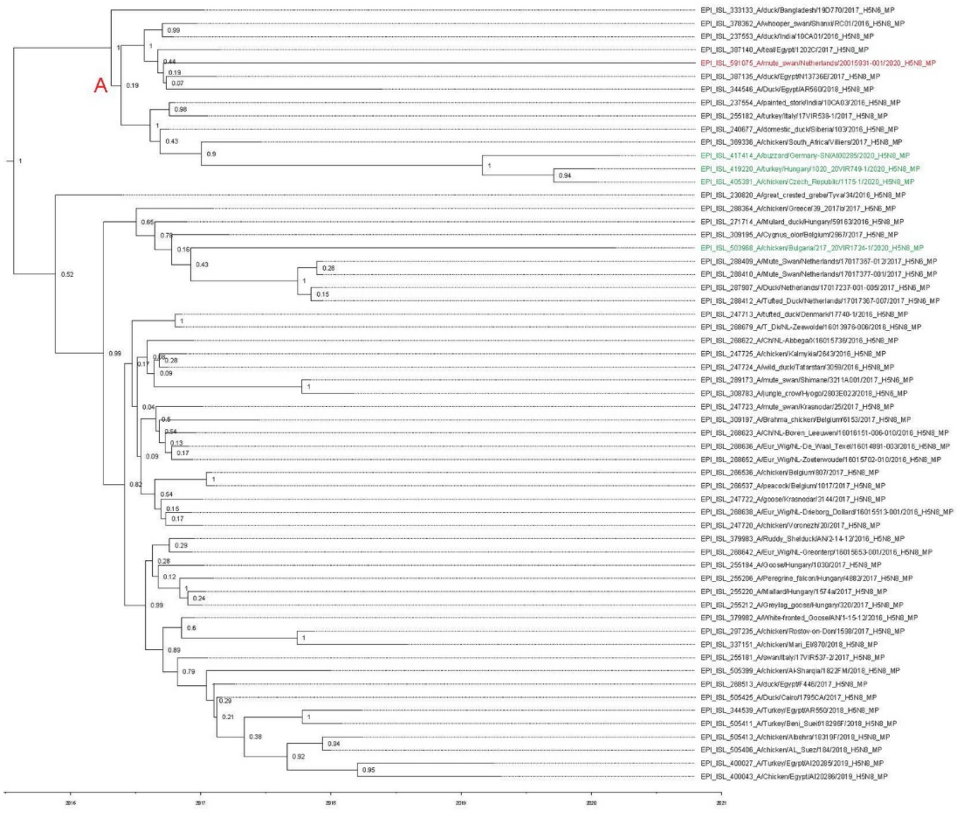
A

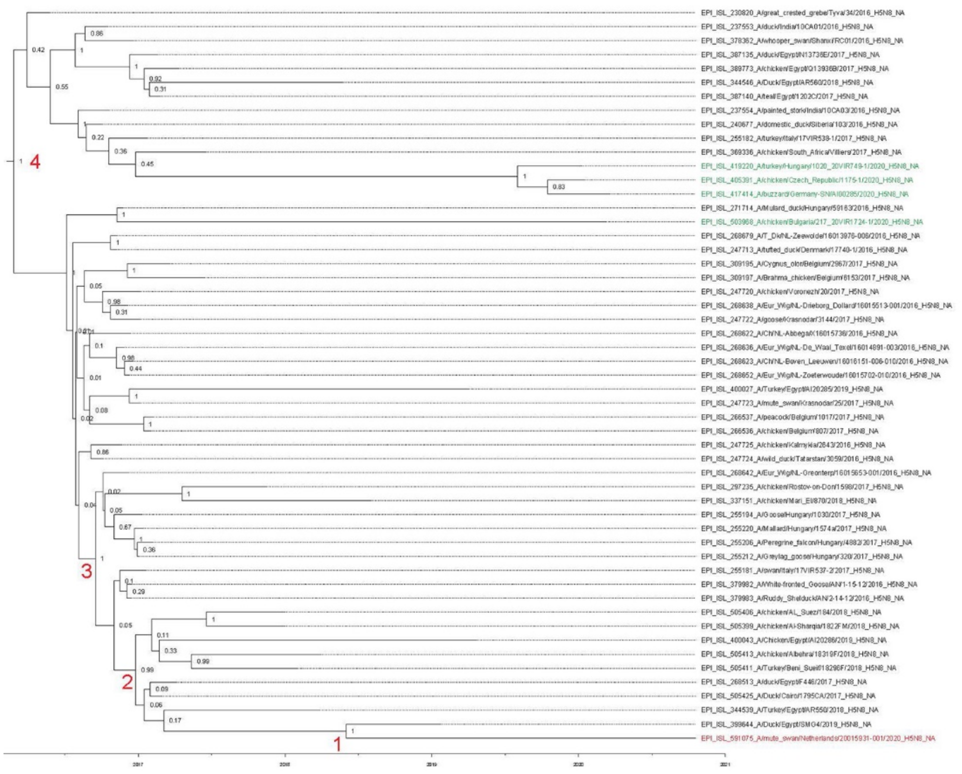
4
3
2
1

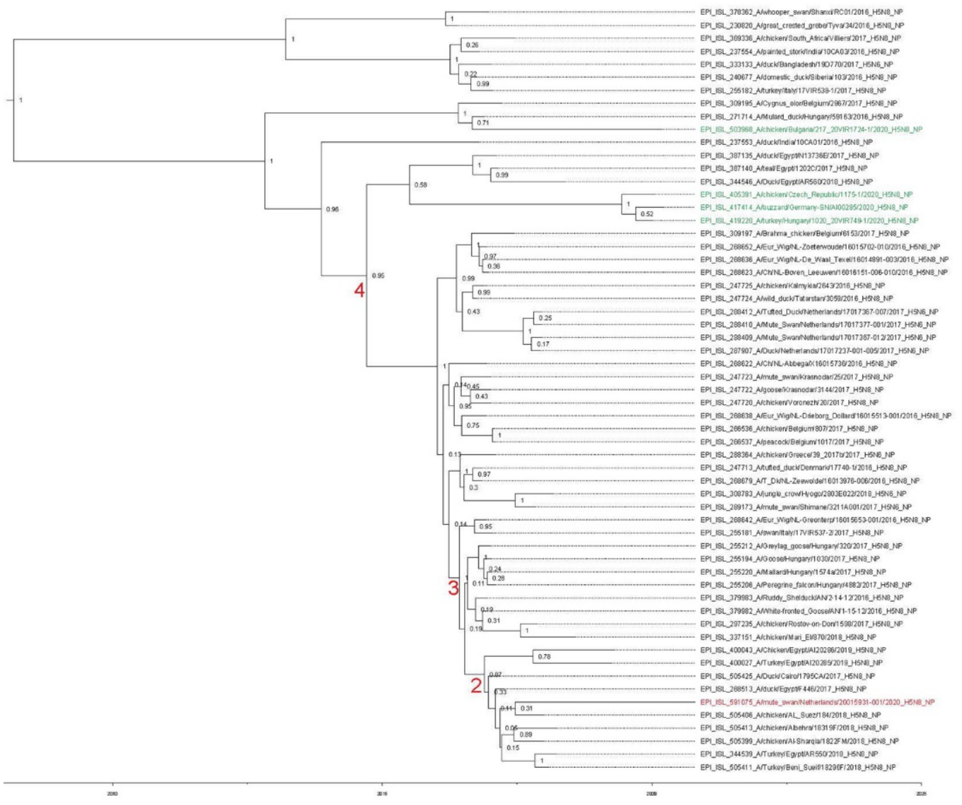

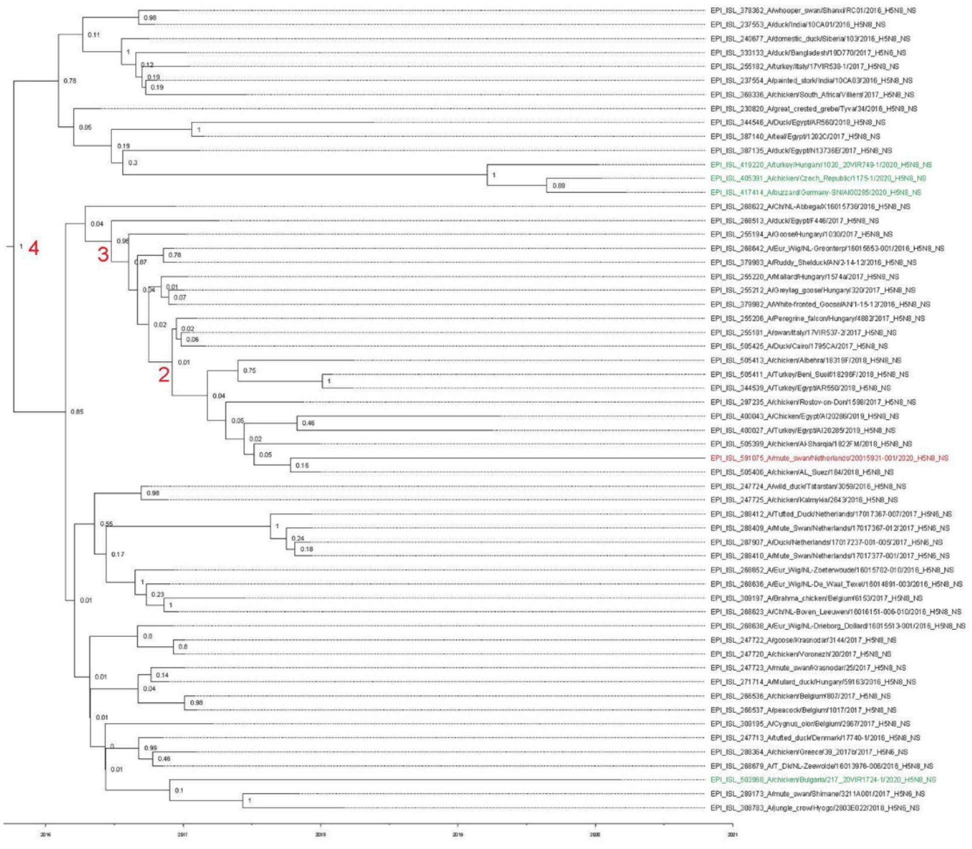

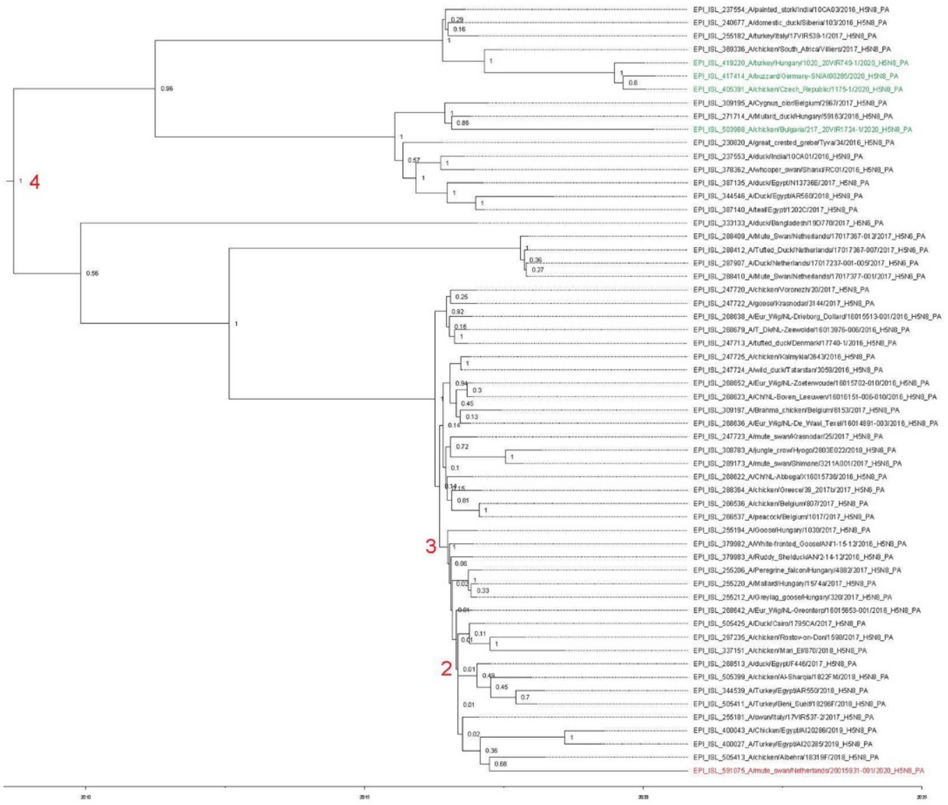
4
3
2

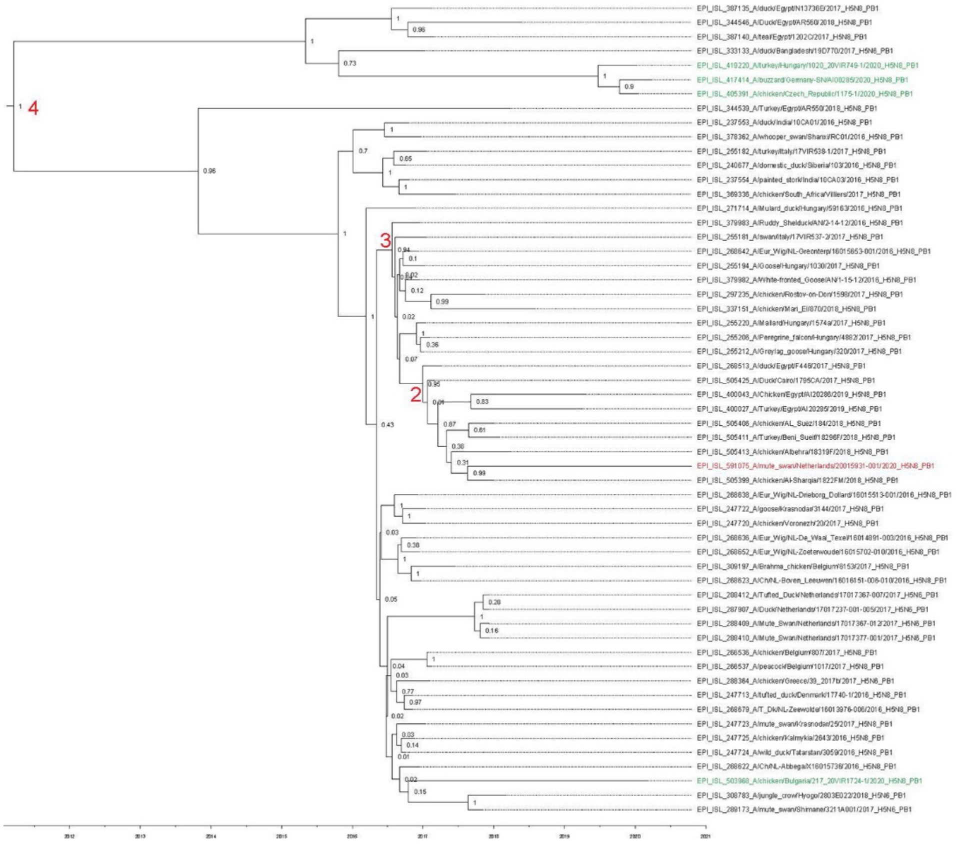
4
3
2

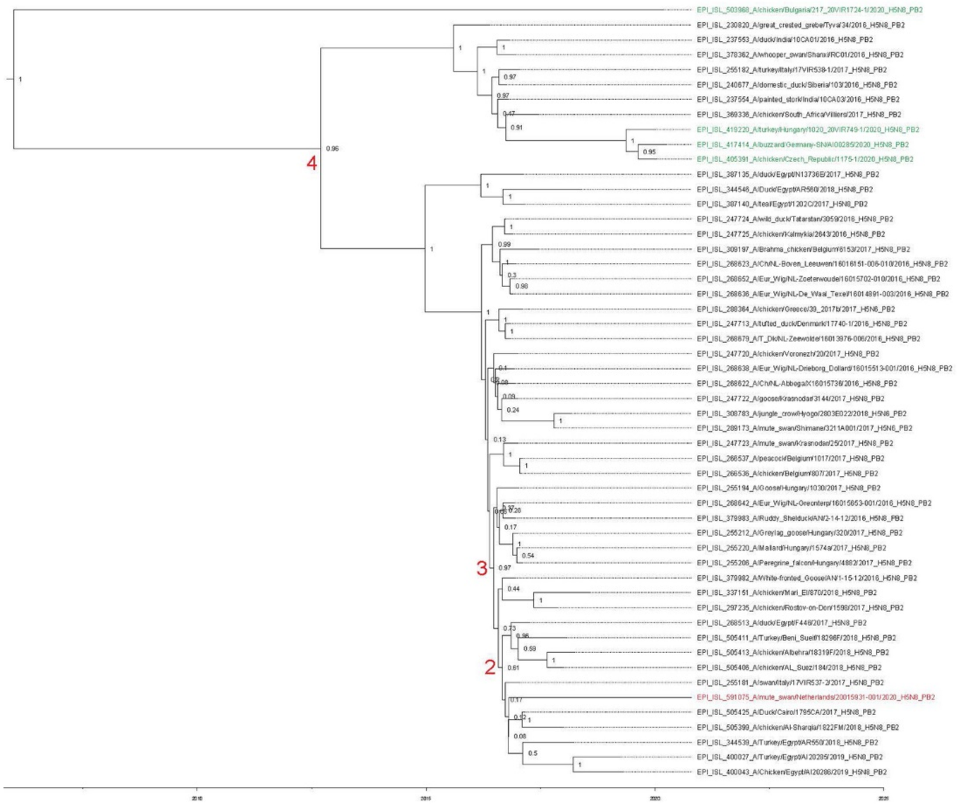

Appendix Figure 2. Molecular dating was performed for the all gene segments. Datasets of maximum-likelihood tree analysis (Appendix Figure1) were used for time-scaled phylogenies, which were reconstructed by using a Bayesian Markov chain Monte Carlo framework implemented in the BEAST software package v 1.10.2 (*5*). Analysis was conducted by using the SRD06 nucleotide substitution model, the Bayesian Skyline coalescent model, and an uncorrelated log normal relaxed molecular clock. Markov chain Monte Carlo runs of 1 x 10^8 states sampling each 1 x 10^4 steps were run to obtain an effective sample size >200. Maximum clade credibility trees were reconstructed with 10% burn-in, and the posterior distribution of relevant parameters were assessed in FigTree v 1.4.4 (*6*). The time to the most recent common ancestor for the numbered nodes is listed in the Table, as is the credible interval and posterior value. For the MP segment, the letter A was used in this figure and the Table to denote the relationship with the viruses found in eastern Europe and Germany during 2020. Because the MP segment was probably introduced by reassortment, this node is not similar to node 4 for the other segments. GISAID accession numbers are shown in the trees (Appendix Table). H5N8 virus isolated in the Netherlands during 2020 is indicated in red; and viruses isolated in eastern Europe, Germany, and Bulgaria during 2020 are indicated in green. Scale bars indicate time intervals. HA, hemagglutinin; MP, matrix protein; NA, neuraminidase; NP, nucleoprotein; NS, nonstructural protein; PA, polymerase acidic, PB1, polymerase basic 1; PB2, polymerase basic 2; 4, node 4.

References

1. Shu Y, McCauley J. GISAID: global initiative on sharing all influenza data. Euro Surveill. 2017;22:30494.

2. Fu L, Niu B, Zhu Z, Wu S, Li W. CD-HIT: accelerated for clustering the next-generation sequencing data. Bioinformatics. 2012;28:3150–2. PubMed https://doi.org/10.1093/bioinformatics/bts565

3. Katoh K, Standley DM. MAFFT multiple sequence alignment software version 7: improvements in performance and usability. Mol Biol Evol. 2013;30:772–80. PubMed https://doi.org/10.1093/molbev/mst010

4. Stamatakis A. RAxML version 8: a tool for phylogenetic analysis and post-analysis of large phylogenies. Bioinformatics. 2014;30:1312–3. PubMed https://doi.org/10.1093/bioinformatics/btu033

5. Suchard MA, Lemey P, Baele G, Ayres DL, Drummond AJ, Rambaut A. Bayesian phylogenetic and phylodynamic data integration using BEAST 1.10. Virus Evol. 2018;4:vey016. PubMed https://doi.org/10.1093/ve/vey016

6. Rambaut A. 2018. FigTree v1.4.4 [cited 2021 Apr 9]. https://github.com/rambaut/figtree/releases
